# 

**Published:** 1924-12

**Authors:** 


### RECENT APPOINTMENTS.

### Medical Officers, &c.

Boul, W. T. G., M.B., Ch.B., Assistant School Medical Officer, East Suffolk; Assistant Medical Officer of Health and Assistant School Medical Officer, Barnsley.

Brough, A. Lothian, M.B., Ch.B.; District Medical Officer for Salford and Broughton areas.

Carse, J., M.B., Ch.B.; Junior Assistant-Medical Officer, Mental Hospital, Gloucestershire.

Conlon, Peter, M.B.; Resident-Assistant Medical Officer, Blackburn.

Davies, S. R. E., M.R.C.S., L.R.C.P., House Surgeon, St. Bartholomew's Hospital; Assistant Medical Officer, Monmouthshire.

Gaunt, Maria L.; Junior Resident Medical Officer, Public Dispensary, Leeds.

Goetze, Marie, L. P., M.R.C.S., L.R.C.P., House Surgeon, General Hospital, Nottingham; Assistant Resident Medical Officer, Central Branch, Royal Infirmary, Manchester.

Hogben, F. A., M.R.C.S., L.R.C.P.; Resident Medical Officer, Royal Infirmary, Manchester.

Jones, William, M.R.C.S., L.R.C.P.; Assistant School Medical Officer and Medical Officer of Health, Rhondda.

Macgillivray, I.M., M.B., Ch.B., Royal Hospital for Sick Children, Edinburgh; Resident Medical Officer, Babies' Hospital, Manchester.

Norwell, Dorothea M., M.B., Ch.B.; Assistant Medical Officer, Perthshire.

Proudfoot, Margaret M., M.B., Ophthalmic House Surgeon, Royal Infirmary, Cardiff; Assistant Medical Officer, Monmouthshire.

Rosenstone, A., M.B., Ch.B.; Resident Medical Officer. Pendlebury Hospital; Assistant Medical Officer, Out-Patients' Department, Royal Manchester Children's Hospital.

Russell, Gladys, M.B.; Assistant Medical Officer, Monmouthshire.

Thompson, Vera, M.B., Ch.B., D.P.H.; Resident Assistant Medical Officer, Howbeck Infirmary, Hartlepool.

Waller, Margaret, M.B.; District Medical Officer and Public Vaccinator, Bradford.

Watts, W. P. T., M.B., B.S.; Resident Medical Officer, Royal Victoria Infirmary, Newcastle.

### Public Health and Hospital Appointments.

Baker, Emmeline; Tuberculosis Health Visitor, Derby.

Barry, Catherine; Matron, Moorlands Hall Home, Dewsbury.

Bell, Elizabeth E.; Health Visitor, Blackburn.

Bishop, A. M.; Matron, Jessop Hospital, Sheffield; Matron, General Hospital, Walsall.

Carr, Miss, A.I.H.A.; Almoner, City of London Hospital for Diseases of the Heart and Lungs, Victoria Park.

Cochrane, M. S.; Matron, Charing Cross Hospital.

Cooke, R. H., M.B., B.S., Senior House Physician, St. Bartholomew's Hospital; Medical Registrar, Hospital for Epilepsy and Paralysis, Maida Vale.

Costain, M.A.; Assistant Matron, Cripple's Guild Holiday Home, Leicester.

Dudfield, Norah W., C.M.B.; Health Visitor, Taunton.

Fillingham, Minnie; Matron, Northern Counties Hospital for Incurables, Manchester.

Griffith, M. H., Assistant Matron, Grove Hospital, Tooting; Matron, Ealing and Chiswick Isolation Hospital.

Higley, D. E. M.; Matron, Cripple's Guild Holiday Home, Leicester.

Howes, Miss, A.I.H.A.; Almoner, Royal Northern Hospital, Holloway Road.

James, Miss, A.I.H.A.; Almoner, St. John's Hospital, Lewisham.

Jones, L. C. A.; Matron, Quantock Lodge Tuberculosis Sanatorium.

Jones, T.; Secretary, Carnarvonshire and Anglesey Infirmary, Bangor.

Pearson, C. M. B.; Health Vistor, Dundee.

Shinnie, A. J., M.D.; Medical Officer of Health, Westminster.

Taggart, L., School Nurse, Oldham; School Nurse, Salford.

Tarrant, A. J. M.; Secretary, Royal London Ophthalmic Hospital.

Williams, S., R.R.C.; Matron, Royal Sussex County Hospital, Brighton.

